# *Vital Signs:* Pregnancy-Related Deaths, United States, 2011–2015, and Strategies for Prevention, 13 States, 2013–2017

**DOI:** 10.15585/mmwr.mm6818e1

**Published:** 2019-05-10

**Authors:** Emily E. Petersen, Nicole L. Davis, David Goodman, Shanna Cox, Nikki Mayes, Emily Johnston, Carla Syverson, Kristi Seed, Carrie K. Shapiro-Mendoza, William M. Callaghan, Wanda Barfield

**Affiliations:** 1 Division of Reproductive Health, National Center for Chronic Disease Prevention and Health Promotion, CDC.

## Abstract

**Background:**

Approximately 700 women die from pregnancy-related complications in the United States every year.

**Methods:**

Data from CDC’s national Pregnancy Mortality Surveillance System (PMSS) for 2011–2015 were analyzed. Pregnancy-related mortality ratios (pregnancy-related deaths per 100,000 live births; PRMRs) were calculated overall and by sociodemographic characteristics. The distribution of pregnancy-related deaths by timing relative to the end of pregnancy and leading causes of death were calculated. Detailed data on pregnancy-related deaths during 2013–2017 from 13 state maternal mortality review committees (MMRCs) were analyzed for preventability, factors that contributed to pregnancy-related deaths, and MMRC-identified prevention strategies to address contributing factors.

**Results:**

For 2011–2015, the national PRMR was 17.2 per 100,000 live births. Non-Hispanic black (black) women and American Indian/Alaska Native women had the highest PRMRs (42.8 and 32.5, respectively), 3.3 and 2.5 times as high, respectively, as the PRMR for non-Hispanic white (white) women (13.0). Timing of death was known for 87.7% (2,990) of pregnancy-related deaths. Among these deaths, 31.3% occurred during pregnancy, 16.9% on the day of delivery, 18.6% 1–6 days postpartum, 21.4% 7–42 days postpartum, and 11.7% 43–365 days postpartum. Leading causes of death included cardiovascular conditions, infection, and hemorrhage, and varied by timing. Approximately sixty percent of pregnancy-related deaths from state MMRCs were determined to be preventable and did not differ significantly by race/ethnicity or timing of death. MMRC data indicated that multiple factors contributed to pregnancy-related deaths. Contributing factors and prevention strategies can be categorized at the community, health facility, patient, provider, and system levels and include improving access to, and coordination and delivery of, quality care.

**Conclusions:**

Pregnancy-related deaths occurred during pregnancy, around the time of delivery, and up to 1 year postpartum; leading causes varied by timing of death. Approximately three in five pregnancy-related deaths were preventable.

**Implications for Public Health Practice:**

Strategies to address contributing factors to pregnancy-related deaths can be enacted at the community, health facility, patient, provider, and system levels.

*On May 7, 2019, this report was posted online as an *MMWR *Early Release.*

## Introduction

Approximately 700 women die annually in the United States from pregnancy-related complications (*1*). Significant racial/ethnic disparities in pregnancy-related mortality exist; black women have a pregnancy-related mortality ratio approximately three times as high as that of white women (*2*,*3*). Better understanding is needed on the circumstances surrounding pregnancy-related deaths and strategies to prevent future deaths.

This report describes the timing and characteristics of pregnancy-related deaths in the United States using 2011–2015 national CDC Pregnancy Mortality Surveillance System (PMSS) data. Data from 13 state maternal mortality review committees (MMRCs) during 2013–2017 were used to determine the percentage of pregnancy-related deaths that were preventable and factors that contributed to the deaths. MMRC-identified strategies for prevention are reported.

## Methods

PMSS was established in 1986 by CDC and the American College of Obstetricians and Gynecologists (ACOG) to evaluate the causes of death and risk factors associated with pregnancy-related deaths. PMSS methodology has been described previously (*2*); CDC’s Division of Reproductive Health requests that all states, the District of Columbia, and New York City send death certificates, linked live birth or fetal death certificates, and additional data when available, on deaths that occurred during pregnancy or within 1 year after delivery. Information on individual deaths are reviewed by medically trained epidemiologists to determine the pregnancy-relatedness and cause (*4*). A death is determined to be pregnancy-related if the death was caused by a pregnancy complication, a chain of events initiated by pregnancy, or the aggravation of an unrelated condition by the physiologic effects of pregnancy. Cause of death coding includes the following 10 mutually exclusive categories: hemorrhage; infection; amniotic fluid embolism; thrombotic pulmonary or other embolism (i.e., air, septic, or fat); hypertensive disorders of pregnancy (i.e., preeclampsia or eclampsia)*; anesthesia complications; cerebrovascular accidents^†^; cardiomyopathy; other cardiovascular conditions (e.g., congenital heart disease, ischemic heart disease, cardiac valvular disease, hypertensive heart disease, and congestive heart failure); and other noncardiovascular medical conditions (e.g., endocrine, hematologic, immunologic, and renal).

Pregnancy-related death data from PMSS for 2011–2015 were analyzed. The pregnancy-related mortality ratio (PRMR) is the number of pregnancy-related deaths per 100,000 live births. PRMRs were calculated by race/ethnicity, age, marital status, education, and year. Birth data, used for determining the number of live births, were obtained from U.S. natality files from the National Center for Health Statistics (*5*). SAS (version 9.4; SAS Institute) was used for all analyses.

Cause and timing of pregnancy-related deaths were analyzed. Timing of death was identified as “during pregnancy” when keywords on the death certificate noted the death was during pregnancy or the pregnancy checkbox option “pregnant at the time of death” was checked. Otherwise, timing of death in relation to the end of pregnancy was determined by comparing date of death on the death certificate with date of live birth or fetal death on linked birth or fetal death certificates. The specific timing of postpartum deaths was classified as unknown if there was no linked birth or fetal death certificate.

Data shared by 13 state MMRCs for deaths that occurred during 2013–2017^§^ were analyzed. Using a standardized data collection system, each multidisciplinary MMRC reviewed available data sources (e.g., medical records, social service records, autopsy reports, and vital records) to determine preventability, factors that contributed to the death, and prevention strategies to address contributing factors. Deaths attributable to suicide, drug overdose, homicide, and unintentional injury were excluded from analyses. MMRCs used the following definition of preventability: “a death is considered preventable if the committee determines that there was some chance of the death being averted by one or more reasonable changes to patient, community, provider, health facility, and/or system factors” (*6*). Percentage of deaths determined by MMRCs to have been preventable were calculated, and chi-squared tests were used to assess whether preventability differed by race/ethnicity or by timing of death. Thematic analyses of MMRC-identified factors that might have contributed to deaths and strategies to prevent future deaths also were conducted.

## Results

During 2011–2015, a total of 3,410 pregnancy-related deaths occurred in the United States; the overall PRMR was 17.2 pregnancy-related deaths per 100,000 live births. The highest PRMRs were in women who were black (42.8) and American Indian/Alaska Native (32.5); these PRMRs were 3.3 and 2.5 times as high, respectively, as were those in white women (13.0) (Table 1). The PRMR was highest among women aged ≥35 years and women who were not married. The overall PRMR fluctuated by year, ranging from 15.9 (2012) to 18.0 (2014).

**TABLE 1:** Pregnancy-related deaths, by sociodemographic characteristics — Pregnancy Mortality Surveillance System, United States, 2011–2015

Characteristic	No. of pregnancy-related deaths	Pregnancy-related mortality ratio*
**Total**	**3,410**	**17.2**
**Race/Ethnicity^†^ (N = 3,400)**		
White	1,385	13.0
Black	1,252	42.8
American Indian/Alaska Native	62	32.5
Asian/Pacific Islander	182	14.2
Hispanic	519	11.4
**Age group (yrs) (N = 3,409)**		
<20	158	11.3
20–24	543	12.1
25–29	751	13.2
30–34	799	15.3
35–39	706	28.7
≥40	452	76.5
**Highest level of education (N = 2,938)**		
Less than high school	572	19.8
High school graduate	1,090	24.2
Some college	775	14.8
College graduate or higher	501	9.4
**Marital status (N = 3,371)**		
Married	1,543	13.1
Not married	1,828	22.8
**Year**		
2011	702	17.8
2012	627	15.9
2013	679	17.3
2014	718	18.0
2015	684	17.2

* Number of pregnancy-related deaths per 100,000 live births.

**^†^** Women identified as white, black, American Indian/Alaska Natives, or Asian/Pacific Islanders were not Hispanic. Hispanic women could be of any race.

When combined, cardiovascular conditions were responsible for >33% of pregnancy-related deaths; these conditions include cardiomyopathy (10.8%), other cardiovascular conditions (15.1%), and cerebrovascular accidents (7.6%). Other leading causes of pregnancy-related death included other noncardiovascular medical conditions (14.3%), infection (12.5%), and obstetric hemorrhage (11.2%). The cause of death could not be determined for 6.7% of pregnancy-related deaths.

Timing of death was known for 2,990 (87.7%) pregnancy-related deaths. Among these deaths, 937 (31.3%) occurred during pregnancy, 506 (16.9%) on the day of delivery, 556 (18.6%) 1–6 days postpartum, 640 (21.4%) 7–42 days postpartum, and 351 (11.7%) 43–365 days postpartum (Table 2). Timing of deaths did not significantly differ between black and white women for most periods; however, a greater proportion of deaths among black women (14.9%) occurred 43–365 days postpartum compared to the proportion of deaths among white women (10.2%) that occurred during the same period (p<0.01).

**TABLE 2:** Pregnancy-related deaths, by cause of death and time of death relative to the end of pregnancy — Pregnancy Mortality Surveillance System, United States, 2011–2015*

Cause of death^†^	Time of death relative to the end of pregnancy^§^					Total no. of deaths
	No. (%) attributed to each cause (row %)					
	During pregnancy	Day of delivery	1–6 days postpartum	7–42 days postpartum	43–365 days postpartum	
Hemorrhage	72 (21.9)	123 (37.4)	105 (31.9)	27 (8.2)	2 (0.6)	**329**
Infection	117 (32.5)	17 (4.7)	83 (23.1)	121 (33.6)	22 (6.1)	**360**
Amniotic fluid embolism	12 (6.9)	114 (65.9)	42 (24.3)	4 (2.3)	1 (0.6)	**173**
Thrombotic pulmonary or other embolism	115 (40.9)	24 (8.5)	41 (14.6)	69 (24.6)	32 (11.4)	**281**
Hypertensive disorders of pregnancy	23 (10.8)	41 (19.3)	94 (44.3)	44 (20.8)	10 (4.7)	**212**
Anesthesia complications	2 (20.0)	3 (30.0)	3 (30.0)	2 (20.0)	0	**10**
Cerebrovascular accidents	68 (29.8)	9 (3.9)	49 (21.5)	79 (34.6)	23 (10.1)	**228**
Cardiomyopathy	48 (15.6)	21 (6.8)	25 (8.1)	75 (24.4)	138 (45.0)	**307**
Other cardiovascular conditions	173 (37.6)	65 (14.1)	61 (13.3)	110 (23.9)	51 (11.1)	**460**
Other noncardiovascular medical conditions	225 (52.7)	61 (14.3)	27 (6.3)	59 (13.8)	55 (12.9)	**427**
Unknown	82 (40.4)	28 (13.8)	26 (12.8)	50 (24.6)	17 (8.4)	**203**
**Total**	**937 (31.3)**	**506 (16.9)**	**556 (18.6)**	**640 (21.4)**	**351 (11.7)**	**2,990**

* Deaths in which timing of death was unknown were excluded (n = 420).

^†^ Cause of death categories are mutually exclusive.

^§^ Time of death might be distant from onset of disease or initial event leading to death.

Distribution of timing of death varied by cause of death (Table 2). Most deaths caused by amniotic fluid embolism occurred on the day of delivery or within 6 days postpartum. Approximately 60% of deaths caused by hypertensive disorders of pregnancy occurred 0–6 days postpartum, whereas those caused by cerebrovascular accidents occurred most frequently 1–42 days postpartum. Deaths caused by cardiomyopathy most commonly occurred 43–365 days postpartum; deaths caused by other cardiovascular conditions occurred most commonly during pregnancy and within 42 days postpartum.

The leading causes of death also varied by time relative to the end of pregnancy. During pregnancy, other noncardiovascular and other cardiovascular conditions were the leading causes of death (Figure); on the day of delivery, hemorrhage and amniotic fluid embolism were the major causes of death. Hemorrhage, hypertensive disorders of pregnancy, and infection were leading causes of death during the first 6 days postpartum. From 6 weeks postpartum (43 days) through the end of the first year (365 days), cardiomyopathy was the leading cause of death.

**FIGURE:**
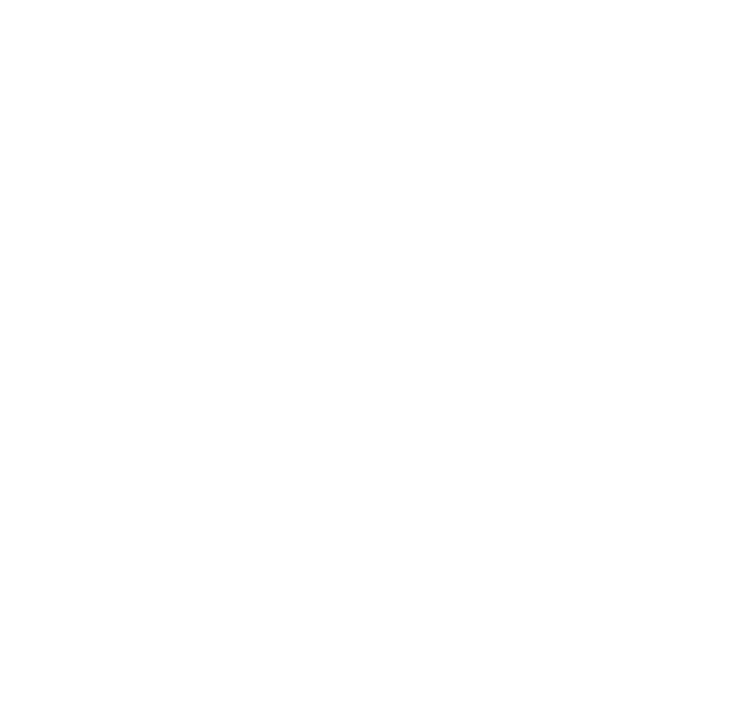
Three most frequent causes of pregnancy-related deaths, by time relative to the end of pregnancy — Pregnancy Mortality Surveillance System, United States, 2011–2015

Among 251 pregnancy-related deaths evaluated for preventability by the 13 MMRCs, a determination was made for 232 (92.4%). Among these, 139 (60.0%) were determined to be preventable deaths. Preventability did not significantly differ between black and white women (p = 0.4), or between Hispanic and white women (p = 0.7), with 57.4% of deaths among black women, 62.7% among white women, and 58.3% among Hispanic women determined to be preventable. Preventability was also similar by timing of pregnancy-related death (59.0% during pregnancy, 53.3% during delivery, 57.1% 1–6 days postpartum, 66.7% 7–42 days postpartum, and 61.9% 43–365 days postpartum; [p = 0.8]).

MMRCs identified an average of three to four contributing factors and two to three prevention strategies per pregnancy-related death. Contributing factors were thematically coded as community factors (e.g., unstable housing and limited access to transportation); health facility factors (e.g., limited experience with obstetric emergencies and lack of appropriate personnel or services); patient factors (e.g., lack of knowledge of warning signs and nonadherence to medical regimens); provider factors (e.g., missed or delayed diagnosis and lack of continuity of care); and system-level factors (e.g., inadequate access to care and poor case coordination) (Table 3). MMRC-identified prevention strategies addressing community factors included expanding clinical office hours and the number of providers who accept Medicaid, prioritizing pregnant and postpartum women for temporary housing programs, and improving access to transportation. Actions addressing health facility factors included implementing obstetric emergency protocols and simulation training, providing telemedicine for facilities without on-site obstetric expertise, and implementing systems to foster communication among multiple providers. Although patient-level contributing factors were commonly identified, prevention strategies to mitigate these factors are often reliant upon providers and health systems. For example, prevention strategies to address patient-level factors included improving patient education materials and providing home health and patient support services. Provider-level prevention strategies included offering provider education to reduce missed or delayed diagnoses, implementing a maternal early warning system (*7*), and improving hand-off communication between obstetricians and other providers. MMRC-identified prevention strategies addressing system-level factors included developing policies to ensure that women deliver at a health facility with an appropriate level of maternal care and extending Medicaid coverage for pregnant women to include 1 year of postpartum care.

**TABLE 3:** Maternal Mortality Review Committee–identified contributing factors and strategies to prevent future pregnancy-related deaths — Maternal Mortality Review Committees, 13 states, 2013–2017

Level	Contributing factor	Strategies to address contributing factor
**Community**	Access to clinical care	Expand office hours, increase number of providers who accept Medicaid, increase availability and use of group prenatal care programs
	Unstable housing	Prioritize pregnant and postpartum women for temporary housing programs
	Lack of, or inadequate, transportation options	Strengthen or build systems to link persons to affordable transportation, or provide vouchers for transport to medical appointments
		Improve availability of transportation services covered by Medicaid
	Obesity and associated chronic disease complications	Improve access to healthy foods and enhance efforts to educate and promote healthy eating habits and weight management strategies
**Health facility**	Limited experience with obstetric emergencies	Implement obstetric emergency simulation training for emergency department and obstetric staff members
		Ensure emergency department staff members ask about recent pregnancy history and consult with obstetrician on call if patient is pregnant or has recently been pregnant
	Lack of appropriate personnel or services	Provide telemedicine for facilities with no obstetric provider on-site
		Ensure Medicaid managed care organizations’ contracts include sufficient access to specialists for patients at high risk
	Lack of guiding protocols or tools to help ensure quality care provision	Ensure sepsis, hemorrhage, and massive transfusion protocols are in place and followed by staff members
		Implement applicable patient safety bundles
		Implement systems to foster communication among multiple providers to ensure proper case coordination
		Implement protocols for using patient navigators
**Patient/Family**	Lack of knowledge of warning signs or need to seek care	Improve counseling and use of patient education materials on warning signs and when to seek care, such as AWHONN Save Your Life discharge instructions
		Implement a public education campaign to increase awareness of signs and symptoms of common complications
	Nonadherence to medical regimens or advice	Standardize patient education to ensure providers relay consistent messages and implement techniques for ensuring patient understanding, such as patient “teaching back” to the provider
		Make education materials available in the clinic and online
		Strengthen and expand access to patient navigators, case managers, and peer support
		Ensure access to interpreter services when needed
		Offer home health or social work follow-up services
**Provider**	Missed or delayed diagnosis	Repeat blood pressure measurement in a timely (and possibly manual) manner when initial blood pressure result is unexpected
		Offer provider education on cardiac conditions in pregnant and postpartum women
		Perform thorough evaluation of patients reporting pain and shortness of breath
	Inappropriate or delayed treatment	Only perform cesarean deliveries when medically indicated
		Implement a maternal early warning system
	Lack of continuity of care	Improve care transition communication among obstetrician-gynecologists and other primary and specialty care physicians
**System**	Inadequate receipt of care	Develop policies to ensure pregnant women are transported to a hospital with an appropriate level of maternal care
		Enlist state perinatal quality collaboratives to identify quality improvement procedures and periodic drills/simulation training for birth facilities, including obstetric emergency drills
		Design education initiatives for emergency department staff members on the care of pregnant and postpartum women
	Case coordination or management	Extend expanded Medicaid coverage eligibility for pregnant women to include 1 year of postpartum care
		Create quality improvement entity to manage outpatient care gaps and improve care coordination
		Implement a postpartum care transition bundle for better integration of services for women at high risk
		Develop procedures for all hospitals to improve documentation of abnormal test results, plan for follow-up care, and management of conditions
		Develop universal health record system that allows for sharing of medical records among hospitals
	Guiding policies, procedures, or standards not in place	Develop protocol for timely referrals and consults
		Ensure all hospitals within a health care system follow the same protocols and policies

**Abbreviation: AWHONN = Association of Women’s Health, Obstetric and Neonatal Nurses**.

## Discussion

Pregnancy-related deaths occur not only during delivery but also during pregnancy and up to 1 year postpartum. The leading causes of pregnancy-related deaths varied by timing of death. Acute obstetric emergencies such as hemorrhage and amniotic fluid embolism most commonly occurred on the day of delivery, whereas deaths caused by hypertensive disorders of pregnancy and thrombotic pulmonary embolism most commonly occurred 0–6 days postpartum, and during pregnancy and 1–42 days postpartum, respectively. Cardiomyopathy was the most common cause of death in the late postpartum period (43–365 days postpartum). The higher proportion of pregnancy-related deaths in the late postpartum period among black women is likely attributable to higher proportion of pregnancy-related deaths due to cardiomyopathy among these women (*8*). Approximately three in five pregnancy-related deaths were determined by MMRCs to be preventable, and preventability did not differ significantly by race/ethnicity or timing of death. Recognizing the major causes of death by timing can help identify opportunities for intervention.

These data demonstrate the need to address the multiple factors that contribute to pregnancy-related deaths during pregnancy, labor and delivery, and postpartum. No single intervention is sufficient; reducing pregnancy-related deaths requires reviewing and learning from each death, improving women’s health, and reducing social inequities across the life span, as well as ensuring quality care for pregnant and postpartum women (*9*). Throughout the preconception, pregnancy, and postpartum periods, providers and patients can work together to optimally manage chronic health conditions (*10*). Standardized approaches to addressing obstetric emergencies can be implemented in all hospitals that provide delivery services. The Alliance for Innovation on Maternal Health (AIM) has provided sets of bundled guidance to provide for such standardization.^¶^ Implementation of this guidance is often supported by perinatal quality collaboratives, state-based initiatives that aim to improve the quality of care for mothers and infants (*11*). Ensuring that pregnant women at high risk for complications receive care in facilities prepared to provide the required level of specialized care also can improve outcomes; professional organizations have developed criteria for recommended levels of maternal care (*12*). CDC has created the Levels of Care Assessment Tool (LOCATe) for public health decision makers to evaluate risk-appropriate care (*13*). In the postpartum period, follow-up care is critical for all women, particularly those with chronic medical conditions and complications of pregnancy (e.g., hypertensive disorders of pregnancy). ACOG recommends that postpartum women have contact with obstetric providers within the first 3 weeks postpartum and recognizes postpartum care as an ongoing process tailored to each woman’s individual needs (*14*).

The findings in this report are subject to at least four limitations. First, errors in reported pregnancy status on the death certificates have been described, potentially leading to overestimation or underestimation of the number of pregnancy-related deaths (*15*). Second, data for specified race or Hispanic-origin groups other than non-Hispanic white and non-Hispanic black should be interpreted with caution because of inconsistencies in reporting these data on death certificates and surveys. Third, generally the pregnancy-relatedness cannot be determined in PMSS for injury deaths such as drug overdoses, suicides, or homicides, or for cancer-related deaths, because of limited information concerning death circumstances. As such, these types of death are often not included in the PRMR. For consistency among data sources, these conditions were not investigated in MMRC data, although MMRC data have found suicides and drug overdoses to be a leading underlying cause of pregnancy-related mortality (*6*). Most (75.0%) of these deaths occur in the late postpartum period. Finally, not all preventable deaths reported by MMRCs had a prevention strategy to address contributing factors; improving quality, completeness, and timeliness of MMRC data can translate into opportunities for prevention. MMRC-identified prevention strategies are based on comprehensive case review by a multidisciplinary group of clinical and nonclinical experts and might not always be drawn from published evidence-based interventions, in part because of a lack of programmatic and policy-based evidence. MMRCs’ access to comprehensive medical and social service records highlights their unique and critical role in understanding all factors contributing to pregnancy-related deaths and using those data to identify strategies to potentially prevent future deaths and contribute to the evidence base.

Pregnancy-related deaths occur during pregnancy, around the time of delivery, and within 1 year postpartum; leading causes of death vary by timing of death. Most pregnancy-related deaths can be prevented. Comprehensive review of pregnancy-related deaths can identify contributing factors and opportunities to implement strategies for preventing future deaths.

**Summary**

**What is already known about this topic?**

Approximately 700 women die annually in the United States from pregnancy-related complications.

**What is added by this report?**

Among pregnancy-related deaths for which timing was known, 31.3% deaths occurred during pregnancy, 16.9% on the day of delivery, 18.6% on days 1–6 postpartum, 21.4% on days 7–42 postpartum, and 11.7% on days 43–365 postpartum. Leading causes of death varied by timing relative to the end of pregnancy. Approximately three in five pregnancy-related deaths were preventable. Contributing factors can be categorized at the community, health facility, patient, provider, and system levels.

**What are the implications for public health practice?**

Most pregnancy-related deaths are preventable, demonstrating the need to identify and implement strategies to address the multiple contributing factors.

## Footnotes

* Deaths caused by hypertension that was not preeclampsia, eclampsia, or gestational hypertension were categorized in the “other cardiovascular conditions” category.

† Deaths caused by cerebrovascular accidents that were a result of preeclampsia or eclampsia were classified in the “hypertensive disorders of pregnancy” category; otherwise, deaths were classified in the “cerebrovascular accidents” category.

§ Arizona (2016), Colorado (2014–2015), Delaware (2013–2017), Florida (2017), Georgia (2013–2014), Hawaii (2015–2016), Illinois (2015), Mississippi (2016–2017), North Carolina (2014–2015), Ohio (2013–2016), South Carolina (2014–2017), Tennessee (2017), and Utah (2015–2016).

¶ https://safehealthcareforeverywoman.org/aim-supported-patient-safety-bundles.

## References

[1] CDC. Pregnancy-related deaths. Atlanta, GA: US Department of Health and Human Services, CDC; 2019. https://www.cdc.gov/reproductivehealth/maternalinfanthealth/pregnancy-relatedmortality.htm

[2] Creanga AA, Syverson C, Seed K, Callaghan WM. Pregnancy-related mortality in the United States, 2011–2013. Obstet Gynecol 2017;130:366–73. 10.1097/AOG.0000000000002114 28697109 PMC5744583

[3] CDC. Pregnancy Mortality Surveillance System. Atlanta, GA: US Department of Health and Human Services, CDC; 2019. https://www.cdc.gov/reproductivehealth/maternalinfanthealth/pregnancy-mortality-surveillance-system.htm

[4] Berg CJ, Callaghan WM, Syverson C, Henderson Z. Pregnancy-related mortality in the United States, 1998 to 2005. Obstet Gynecol 2010;116:1302–9. 10.1097/AOG.0b013e3181fdfb11 21099595

[5] CDC. Birth data. Atlanta, GA: US Department of Health and Human Services, CDC; 2019. https://www.cdc.gov/nchs/nvss/births.htm

[6] Building U.S. Capacity to Review and Prevent Maternal Deaths. Report from nine maternal mortality review committees. Washington, DC: Review to Action; 2018. http://reviewtoaction.org/sites/default/files/national-portal-material/Report%20from%20Nine%20MMRCs%20final_0.pdf

[7] Mhyre JM, D’Oria R, Hameed AB. The maternal early warning criteria: a proposal from the national partnership for maternal safety. Obstet Gynecol 2014;124:782–6. 10.1097/AOG.0000000000000480 25198266

[8] Creanga A, Berg C, Syverson C, Seed K, Bruce F, Callaghan W. Race, ethnicity, and nativity differentials in pregnancy-related mortality in the United States: 1993–2006. Obstet Gynecol 2012;120:261-8. 10.1097/AOG.0b013e31825cb87a 22825083

[9] Lu MC. Reducing maternal mortality in the United States. JAMA 2018;320:1237–8. 10.1001/jama.2018.11652 30208484

[10] Howell EA. Reducing disparities in severe maternal morbidity and mortality. Clin Obstet Gynecol 2018;61:387–99. 10.1097/GRF.0000000000000349 29346121 PMC5915910

[11] Henderson ZT, Ernst K, Simpson KR, The national network of state perinatal quality collaboratives: a growing movement to improve maternal and infant health. J Womens Health 2018;27:221–6. 10.1089/jwh.2018.6941 PMC11009782 29634446

[12] American College of Obstetricians and Gynecologists; Society for Maternal-Fetal Medicine; Menard MK, Kilpatrick S, Saade G, Levels of maternal care. Am J Obstet Gynecol 2015;212:259–71. 10.1016/j.ajog.2014.12.030 25620372

[13] CDC. CDC Levels of Care Assessment Tool (CDC LOCATe). Atlanta, GA: US Department of Health and Human Services, CDC; 2019. https://www.cdc.gov/reproductivehealth/maternalinfanthealth/LOCATe.html

[14] American College of Obstetricians and Gynecologists. ACOG committee opinion no. 736: optimizing postpartum care. Obstet Gynecol 2018;131:e140–50. 10.1097/AOG.0000000000002633 29683911

[15] Baeva S, Saxton DL, Ruggiero K, Identifying maternal deaths in Texas using an enhanced method, 2012. Obstet Gynecol 2018;131:762–9. 10.1097/AOG.0000000000002565 29630012

